# Drug-induced hypersensitivity syndrome induced by propylthiouracil: case report and literature review

**DOI:** 10.1186/s13223-022-00707-w

**Published:** 2022-08-06

**Authors:** Fang Wu, Ting Jin, Chengxin Shang, Xihua Lin, Xiaoqin Gong, Zhou Wang

**Affiliations:** 1 Department of Endocrinology, Zhejiang University School of Medicine Sir Run Run Shaw Hospital, Hangzhou, Zhejiang China; 2 Biomedical Research Center and Key Laboratory of Biotherapy of Zhejiang Province, Sir Run Run Shaw Hospital, School of Medicine, Zhejiang University, Hangzhou, Zhejiang China; 3 Department of Gynecology, Pujiang People Hospital, Pujiang Country, Zhejiang China

**Keywords:** Drug-induced hypersensitivity syndrome, Propylthiouracil, Human leukocyte antigen

## Abstract

**Background:**

Drug-induced hypersensitivity syndrome (DIHS) is a rare, potentially life-threatening systemic drug reaction. Antithyroid drugs (ATDs) causing DIHS have seldom been reported before.

**Case presentation:**

We present a case of propylthiouracil (PTU)-induced DIHS, which included fever, skin rash, lymphadenopathy, hepatosplenomegaly, serious liver and kidney dysfunction, peripheral blood eosinophilia, and atypical lymphocytosis. Following supportive therapy, intravenous immunoglobulin (IVIG), and systemic corticosteroid, the patient experienced a resolution of fever and rash combined with progressive normalization of hematological index and organ function. These clinical features, and the skin lesion biopsy confirmed DIHS diagnosis.

**Conclusions:**

To our knowledge, this is the second reported case of PTU-induced DIHS worldwide and the first human leukocyte antigen (HLA) typing of PTU-induced DIHS. Clinicians should cautiously distinguish hyperthyroidism etiology and identify the indication of ATDs. Timely recognition and formal DIHS treatment are required in patients with ATDs.

## Background

Drug-induced hypersensitivity syndrome (DIHS) is a severe adverse drug reaction characterized by extensive acute skin lesions, fever, lymphadenopathy, hematological abnormalities, and multiple organ involvement. There is currently no gold standard for DIHS diagnosis. The recognized diagnostic criteria are proposed by the Japanese Research Committee on Severe Cutaneous Adverse Reaction (J-SCAR) and the European Registry of Severe Cutaneous Adverse Reaction (RegiSCAR) [1, 2]. Although this syndrome is rare, it is life-threatening, with a reported mortality of 10–20% [3]. Therefore, early recognition and prompt treatment are essential for DIHS prognosis. Antithyroid drugs (ATDs) are the effective treatment option for Graves' hyperthyroidism, including methimazole (MMI) and propylthiouracil (PTU). Here, we report a case of DIHS induced by PTU for hyperthyroid disease.

## Case presentation

A 24-year-old female patient presented with menstrual irregularity (hypomenorrhea and menstrual phase shortening), hand tremor, hyperorexia and goiter for one year. Due to high free triiodothyronine (FT_3_: 15.25 pmol/L, 3.1–6.80 pmol/L) and free thyroxine (FT_4_: 71 pmol/L, 12.00–22.00 pmol/L) and low thyroid-stimulating hormone (TSH: 0.01uIU/ml, 0.27–4.20uIU/ml), she was diagnosed with hyperthyroidism four months ago at the local hospital. Without further thyroid-stimulating hormone receptor antibody (TRAb) and thyroid ultrasound examinations, PTU at a dose of 100 mg three times daily was administered. She had no other significant medical history or known allergies.

About three weeks later, she presented with a temperature of 39–40 ℃ and a diffuse, itchy, maculopapular rash. After terminating PTU and adding anti-allergy treatment, the symptoms subsided, and the skin rash was desquamated. However, she had a fever again (38.8 ℃) one month later, along with nausea, vomiting, diarrhea and systemic oedema. On admission to a provincial hospital, her hematological abnormalities were presented with increased eosinophils ratio (eosinophils: 6.4%, 0.2–5.0%; neutrophils: 58.3%, 40.3–72.3%; lymphocytes: 26.2%, 20.4–51.0%; basophils: 0.2%, 0.0–1.0%) and progressed to elevated monocyte ratio (15.6%, 3.0–12.0%), increased atypical cells (13%, 0–0%), decreased hemoglobin (80 g/L, 110–150 g/L), and blood platelets (53*10^9^ cells/L, 100–300*10^9^ cells/L). Laboratory examinations revealed the liver injury (alanine amino transferase 199 U/L, 3–50 U/L) and kidney injury (creatinine 227 µmol/L, 44–133 µmol/L). A thyroid panel showed decreased FT_3_, normal FT_4_, and decreased TSH. To stabilize the patient, supportive therapies were adminstered, including hepatic function protection and hemodialysis. Fever was uncontrolled despite intravenous immunoglobulin (IVIG, 10 g/d for 3 days). After nine days of intravenous methylprednisolone (40 mg/d), the skin rash and oedema gradually subsided. Additionally, her body temperature, liver and kidney functions returned to normal. However, she experienced a recrudescence of symptoms after stopping corticosteroid treatment.

**Fig. 1:**
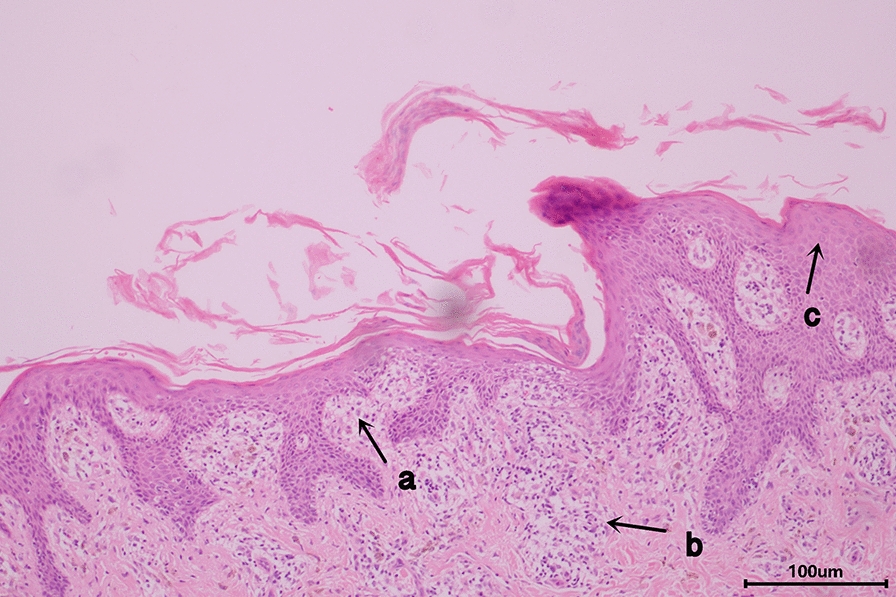
Haematoxylin and eosin staining of the skin biopsy. Finding from the skin biopsy: dermal lymphocytic infiltrate and occasional perivascular monocytic infiltrate, with the presence of interface vacuolization (**a**), perifollicular lymphocytic infiltration (**b**), epidermal spongiosis (**c**). Hematoxylin and eosin stain; original magnification: X200

Two weeks later, the patient was admitted to our hospital. Immunoglobulin G (IgG) concentrations were found to be high at 2890 mg/dL (694–1620 mg/dL). The immunoglobulin M (IgM) tests on peripheral blood sample were negative for coxsackievirus, Epstein-Barr virus (EBV), and cytomegalovirus (CMV). Human herpes virus-6 (HHV-6) DNA was not detected in serum by polymerase chain reaction. Thyroid scintigraphy revealed a heterogeneous low uptake of bilateral thyroid gland (total Tc99m uptake was 0.3% at 30 min). The pathological features of skin lesions showed numerous dermal lymphocytic, and occasional perivascular monocytic infiltrates, with the presence of interface vacuolization, perifollicular lymphocytic infiltration, and epidermal spongiosis (Fig. 1).

Following a multidisciplinary team discussion, this patient was diagnosed with DIHS, which was most likely triggered by PTU. Her hyperthyroid condition was caused by Hashimoto's thyroiditis. During hospitalization, she had no fever, and her rash gradually resolved. We treated her only with mucopolysaccharide polysulfate cream applied to her skin lesions, along with metoprolol to control her heart rate.

Within one month after discharge (four months after discontinuing PTU), the patients developed to subclinical hypothyroidism. On follow-up one month later (five months after discontinuing PTU), she progressed to overt hypothyroidism and was received levothyroxine replacement therapy. During follow-up, her thyroid function returned to normal with levothyroxine therapy (Table 1).

**Table 1:** Follow-up of thyroid function in different hospitals

Items	before PTU	1 month after stopping PTU	3 month after stopping PTU	4 months after stopping PTU	5 months after stopping PTU	11 months after stopping PTU
Hospital	A	B	C	C	C	C
TSH	0.01	0.07	0	6.81	34.93	1.16
	0.27–4.20uIU/ml	0.34–5.6uIU/L	0.35–4.94mIU/L			
FT_3_	15.25	3.5	6.55			
	3.1–6.80 pmol/L	3.8–6.0 pmol/L	1.71–3.71 pg/ml			
FT_4_	71	16.2	2.23			
	12.00–22.00 pmol/L	7.9–17.1 pmol/L	0.70–1.48 ng/dl			
TT_3_	5.46	0.94	2.42	0.86	0.76	1.32
	1.3–3.10 nmol/L	1.05–2.73 nmol/L	0.57–1.59 ng/ml			
TT_4_	239.7	99.79	13.06	4,75	3.68	8.88
	66.00–181.00 nmol/L	78.38–170.0 nmol/L	4.87–11.72ug/dl			

A: local hospital; B: provincial hospital; C: our hospital

*FT3* free triiodothyronine, *FT4* free thyroxine, *PTU* propylthiouracil, *TSH* thyroid stimulating hormone, *TT3* total triiodothyronine, *TT4* total thyroxin

The high-resolution of human leukocyte antigen (HLA) typing was retrospectively assayed using sequence-based typing method, as presented in Table 2.

**Table 2:** HLA typing of the patient

Gene	HLA-A*	HLA-B*	HLA-C*	HLA-DRB1*	HLA-DQB1*	HLA-DPB1*
Allele	02:02, 11:01	13:01, 46:01	01:02, 03:04	04:05, 08:03	06:01, 04:01	02:02, 02:02

*HLA* human leukocyte antigen

## Discussion and conclusions

DIHS is a severe drug reaction characterized by cutaneous manifestations and multiple organ involvement. It often begins with a fever above 38 ℃, shortly followed by maculopapular rash or erythema multiforme. Typically, the skin lesion originated on the face, upper torso, or upper limbs and spreads to the whole body, occasionally accompanied by edema in eyelid, face, or neck [4, 5]. In severe cases, exfoliative dermatitis or erythroderma develop. Besides skin involvement, hepatosplenomegaly can be present and is frequently accompanied by liver and/or kidney injury during illness. There can be marked hematologic abnormalities, including leukocytosis (> 11*10^9^ cells/L), atypical lymphocytosis (> 5%), and eosinophilia (> 1.5*10^9^ cells/L). Serum immunoglobulin levels, including IgG, immunoglobulin A (IgA), and IgM, decreased at onset and reached their lowest levels several days after cessation of offending drugs. Subsequently, serum IgG level increases significantly after 1–2 weeks and returns to normal upon complete disease remission [6]. DIHS has a polymorphous dermatopathological appearance. A dense superficial and/or perivascular lymphocytic infiltrate is commonly present, sometimes accompanied by eosinophils, neutrophils, or extravasated erythrocytes [7]. In addition, the spongiosis or acanthosis in the epidermis was frequently consistent [8, 9]. Occasionally, a sufficiently dense lymphocytic infiltrate or the presence of atypical lymphocytes mimics lymphoma [10, 11]. Other histopathological features include interface vacuolization, lichenoid infiltrate, and individually apoptotic keratinocytes [12]. DIHS usually occurs three weeks to three months after offending drug administration, later than most other drug reactions [1]. However, symptoms may prolong and relapse even after withdrawal of offending drug [13]. Multiple organ involvement differentiates DIHS from common drug eruptions, including lymphatic, hepatic, nephritic, cardiac, neurologic, and endocrine systems [14]. Furthermore, severe hepatitis is the main cause of mortality. Due to multiple organ abnormalities, DIHS may be misdiagnosed for acute infectious diseases, lymphoproliferative diseases, and autoimmune diseases.

The absence of polyneuropathy and polyneuropathy ruled out POEMS syndrome. And, Castleman disease was unlikely due to the absence of pathologic manifestation of lymph nodes and bone marrow.

Although DIHS pathogenesis remains ambiguous, it is generally regarded as a hypersensitive reaction and sequential reactivation of HHV-6. Typical DIHS exhibits a bimodal clinical course, with the first stage including drug hypersensitivity reaction and the second stage involving reactivation of HHV-6 [1, 10]. Additionally, EBV, CMV, and HHV-7 are reactivated during the course of DIHS [15, 16]. Both Hashimoto and Shiohara groups investigated HHV-6 reactivation in severe cutaneous adverse drug reactions, detecting HHV-6 DNA only in DIHS [16, 17]. As a result, J-SCAR group established all seven diagnostic criteria for DIHS, including HHV-6 reactivation [1]. In this patient, HHV-6 DNA and IgM antibody for coxsackievirus, EBV and CMV, were not detected, possibly due to inappropriate sampling timing following virus reaction.

Several evidences have demonstrated that genetic factors are implicated with DIHS, particularly specific HLAs subtypes [18]. HLA pre-screening has been shown to reduce the risk of DIHS in selceted populations when medications such as carbamazepine, allopurinol and abacavir are employed. We retrospectively summarize recent progresses in identifying pharmacogenetic associations with DIHS in different populations (Table 3) [19]. A systematic meta-analysis of pharmacogenomics studies by Chi et al. revealed that HLA-B*27:05, HLA-B*38:02, and HLA-DRB1*08:03 alleles were linked to ATD-induced agranulocytosis, particularly in MMI [20]. Additionally, our case possessed HLA-DRB1*08:03 allele. However, large-scale studies of ATD-induced DIHS must assess the possible genetic association with HLA. 3D structural modeling was conducted to observe interactions between HLA proteins and ATD drugs [21]. In the pocket of HLA-DRB1*08:03, one asparagine residue was predicted to stabilize ATD drug molecules by bonding with sulfur and nitrogen atoms of the drug. However, the detailed mechanisms underlying ATD-HLA complex and the determinants of DIHS response remain unknown. Additional experiments are required.

**Table 3:** Pharmacogenetics of HLA-associated DIHS

Associated drug		HLA allele	Ethnicity
Aromatic anticonvulsants	Carbamazepine	A*31:01	Northern European, Japanese, Korean
	Phenytoin	B*51:01	Thai
		B*15:13	Malaysian
	Lamotrigine	A*24:02	Spanish
Uric-acid-lowering drug	Allopurinol	B*58:01	Han Chinese, Korean, Japanese, Thai, European
Antriretroviral drug	Abacavir	B*57:01	European, African
	Nevirapine	C*04	African, Han Chinese
		C*08 or C*08-B*14 haplotype	Italian, Japanese
Antibiotics	Dapsone	B*13:01	Han Chinese

*DIHS* drug-induced hypersensitivity syndrome, *HLA* human leukocyte antigen

Systemic corticosteroid therapy is currently the most common treatment for DIHS. Systemic steroid therapy is often administered at a dose of 40–60 mg/d of prednisolone or equivalent. The dosage should then be tapered over 6–8 weeks to prevent relapse or organic damage [13]. When the aforementioned steroid therapy falis to relieve or exacerbates symptoms, patients may be treated with pulsed intravenous methylprednisolone (30 mg/kg for 3 days), IVIG, plasmapheresis, or a combination of the above therapies [22]. The patient, in this case, was treated with an insufficient course of steroid therapy (equivalent to intravenous prednisolone, 50 mg/d for nine days), which resulted in a repeated outbreak and protracted course of the disease. However, possibly due to a less serious condition and combined treatment with IVIG, the patient recovered. IVIG contains anti-virus IgG, which could work against HHV-6 [23]. IgG has been reported to block Fas–Fas ligand-mediated keratinocytes apoptosis [24]. Additionally, IgG has an anti-inflammatory function by increasing glucocorticoid receptor sensitivity to inhibit lymphocyte activation [25].

Until now, clinical cases of DIHS following ATD treatment have so far rarely been reported [26, 27]. We identified three cases (our case and two reported cases) on DIHS with ATDs (Table 4). Implicated drugs included MMI (n = 1) and PTU (n = 2). All three patients had a fever and diffused maculopapular rash. In the case reports, common clinical features included hematologic abnormality, liver abnormality, and lymphadenopathy. Ozaki et al. observed an increase in the titer of HHV-6 IgG and IgM antibodies, implying HHV-6 reactivation [26]. The mainstay of treatment in the three patients was corticosteroid therapy.

**Table 4:** Reported cases of ATDs-induced DIHS

Study	Ozaki (2005)	Ye (2010)	Our study
Age	50 years	34 years	24 years
Sex	Male	Female	Female
ATDs	MMI	PTU	PTU
Time of onset after ATDs	1.5 months	6 weeks	3 weeks
Fever	Yes	Yes	Yes
Rash	Yes	Yes	Yes
Hematologic abnormality			
Leucocytosis	Yes	NR	No
Atypical lymphocytosis	No	NR	Yes
Eosinophilia	NR	Yes	Yes
Liver abnormality	Yes	Yes	Yes
Other affected organs	Lung	NR	Kidney
Lymphademopathy	NR	Yes	Yes
HHV-6 detection	Yes	NR	No
Other virus detection	CMV	NR	No
Treatment	Corticosteroid	Corticosteroid	Corticosteroid, IVIG

*ATDs* antithyroid drugs, *DIHS* drug-induced hypersensitivity syndrome, *HHV-6* human herpes virus-6, *IVIG* intravenous immunoglobulin, *MMI* methimazole, *NR* not reported, *PTU* propylthiouracil

There are two important lessons to be learned from our case. First, when treating hyperthyroidism with PTU, physicians should be vigilant for DIHS possibility, particularly late-onset drug eruption with fever, lymphadenopathy, hematological abnormalities, and multiple organ involvement. In our case, failure to promptly diagnose and initiate formal treatment resulted in symptom relapse. IgM, IgG, and DNA testing for HHV-6 has a certain clinical utility. According to condition severity, timely administration of effective treatment is critical for reducing mortality rates of DIHS. Second, the patient with the first examination exhibited Hashimoto's thyroiditis, which progressed to hyperthyroidism rather than Graves' hyperthyroidism. In general, most patients who develop Hashimoto's thyroiditis with hyperthyroidism do not require ATDs treatment. To avoid unnecessary adverse drug reactions, a cautious differential diagnosis of hyperthyroidism etiology is extremely necessary.

## Data Availability

The data analyzed for the case report are available from the corresponding author upon reasonable request and with permission of the legal guardian of the patient.

## Abbreviations

DIHS: Drug-induced hypersensitivity syndrome
ATDs: Antithyroid drugs
PTU: Propylthiouracil
IVIG: Intravenous immunoglobulin
HLA: Human leukocyte antigen
J-SCAR: Japanese Research Committee on Severe Cutaneous Adverse Reaction
RegiSCAR: European Registry of Severe Cutaneous Adverse Reaction
FT_3_: Free triiodothyronine
FT_4_: Free thyroxine
TSH: Thyroid-stimulating hormone
TRAb: Thyroid-stimulating hormone receptor antibody
IgG: Immunoglobulin G
IgM: Immunoglobulin M
EBV: Epstein-Barr virus
CMV: Cytomegalovirus
HHV-6: Human herpes virus-6

## References

[1] Shiohara T, Iijima M, Ikezawa Z, Hashimoto K (2007). The diagnosis of a DRESS syndrome has been sufficiently established on the basis of typical clinical features and viral reactivations. Br J Dermatol.

[2] Peyriere H, Dereure O, Breton H, Demoly P, Cociglio M, Blayac JP (2006). Variability in the clinical pattern of cutaneous side-effects of drugs with systemic symptoms: does a DRESS syndrome really exist?. Br J Dermatol.

[3] Botelho LF, Higashi VS, Padilha MH, Enokihara MM, Porro AM (2012). DRESS: clinicopathological features of 10 cases from an University Hospital in Sao Paulo. An Bras Dermatol.

[4] Jeung YJ, Lee JY, Oh MJ, Choi DC, Lee BJ (2010). Comparison of the causes and clinical features of drug rash with eosinophilia and systemic symptoms and stevens-johnson syndrome. Allergy Asthma Immunol Res.

[5] Kardaun SH, Sekula P, Valeyrie-Allanore L, Liss Y, Chu CY, Creamer D (2013). Drug reaction with eosinophilia and systemic symptoms (DRESS): an original multisystem adverse drug reaction. Results from the prospective RegiSCAR study. Br J Dermatol.

[6] Kano Y, Seishima M, Shiohara T (2006). Hypogammaglobulinemia as an early sign of drug-induced hypersensitivity syndrome. J Am Acad Dermatol.

[7] Skowron F, Bensaid B, Balme B, Depaepe L, Kanitakis J, Nosbaum A (2015). Drug reaction with eosinophilia and systemic symptoms (DRESS): clinicopathological study of 45 cases. J Eur Acad Dermatol Venereol.

[8] Walsh S, Diaz-Cano S, Higgins E, Morris-Jones R, Bashir S, Bernal W (2013). Drug reaction with eosinophilia and systemic symptoms: is cutaneous phenotype a prognostic marker for outcome? A review of clinicopathological features of 27 cases. Br J Dermatol.

[9] Chi MH, Hui RC, Yang CH, Lin JY, Lin YT, Ho HC (2014). Histopathological analysis and clinical correlation of drug reaction with eosinophilia and systemic symptoms (DRESS). Br J Dermatol.

[10] Bocquet H, Bagot M, Roujeau JC (1996). Drug-induced pseudolymphoma and drug hypersensitivity syndrome (Drug Rash with Eosinophilia and Systemic Symptoms: DRESS). Semin Cutan Med Surg.

[11] Roujeau JC (2005). Clinical heterogeneity of drug hypersensitivity. Toxicology.

[12] Cho YT, Liau JY, Chang CY, Yang CW, Chen KL, Chen YC (2016). Co-existence of histopathological features is characteristic in drug reaction with eosinophilia and systemic symptoms and correlates with high grades of cutaneous abnormalities. J Eur Acad Dermatol Venereol.

[13] Shiohara T, Mizukawa Y (2019). Drug-induced hypersensitivity syndrome (DiHS)/drug reaction with eosinophilia and systemic symptoms (DRESS): An update in 2019. Allergol Int.

[14] Husain Z, Reddy BY, Schwartz RA (2013). DRESS syndrome: part I. Clinical perspectives. J Am Acad Dermatol.

[15] Kano Y, Hiraharas K, Sakuma K, Shiohara T (2006). Several herpesviruses can reactivate in a severe drug-induced multiorgan reaction in the same sequential order as in graft-versus-host disease. Br J Dermatol.

[16] Ishida T, Kano Y, Mizukawa Y, Shiohara T (2014). The dynamics of herpesvirus reactivations during and after severe drug eruptions: their relation to the clinical phenotype and therapeutic outcome. Allergy.

[17] Tohyama M, Hashimoto K, Yasukawa M, Kimura H, Horikawa T, Nakajima K (2007). Association of human herpesvirus 6 reactivation with the flaring and severity of drug-induced hypersensitivity syndrome. Br J Dermatol.

[18] Cheng CY, Su SC, Chen CH, Chen WL, Deng ST, Chung WH (2014). HLA associations and clinical implications in T-cell mediated drug hypersensitivity reactions: an updated review. J Immunol Res.

[19] Chen CB, Abe R, Pan RY, Wang CW, Hung SI, Tsai YG (2018). An Updated review of the molecular mechanisms in drug hypersensitivity. J Immunol Res.

[20] Chen WT, Chi CC (2019). Associations of HLA genotypes with antithyroid drug-induced agranulocytosis: a systematic review and meta-analysis of pharmacogenomics studies. Br J Clin Pharmacol.

[21] Chen PL, Shih SR, Wang PW, Lin YC, Chu CC, Lin JH (2015). Genetic determinants of antithyroid drug-induced agranulocytosis by human leukocyte antigen genotyping and genome-wide association study. Nat Commun.

[22] Shiohara T, Inaoka M, Kano Y (2006). Drug-induced hypersensitivity syndrome (DIHS): a reaction induced by a complex interplay among herpesviruses and antiviral and antidrug immune responses. Allergol Int.

[23] Kito Y, Ito T, Tokura Y, Hashizume H (2012). High-dose intravenous immunoglobulin monotherapy for drug-induced hypersensitivity syndrome. Acta Derm Venereol.

[24] Scheuerman O, Nofech-Moses Y, Rachmel A, Ashkenazi S (2001). Successful treatment of antiepileptic drug hypersensitivity syndrome with intravenous immune globulin. Pediatrics.

[25] Kano Y, Inaoka M, Sakuma K, Shiohara T (2005). Virus reactivation and intravenous immunoglobulin (IVIG) therapy of drug-induced hypersensitivity syndrome. Toxicology.

[26] Ozaki N, Miura Y, Sakakibara A, Oiso Y (2005). A case of hypersensitivity syndrome induced by methimazole for Graves' disease. Thyroid.

[27] Ye YM, Kim JE, Kim JH, Choi GS, Park HS (2010). Propylthiouracil-induced DRESS syndrome confirmed by a positive patch test. Allergy.

